# Highly Sensitive Determination of Tenofovir in Pharmaceutical Formulations and Patients Urine—Comparative Electroanalytical Studies Using Different Sensing Methods

**DOI:** 10.3390/molecules27061992

**Published:** 2022-03-19

**Authors:** Natalia Festinger, Kaja Spilarewicz-Stanek, Kamila Borowczyk, Dariusz Guziejewski, Sylwia Smarzewska

**Affiliations:** 1 Łukasiewicz Research Network-Textile Research Institute, 5/15 Brzezinska St., 92-103 Lodz, Poland; natalia.festinger@iw.lukasiewicz.gov.pl; 2 Faculty of Chemistry, Jagiellonian University, 2 Gronostajowa St., 30-387 Krakow, Poland; kaja.spilarewicz-stanek@uj.edu.pl; 3 Department of Environmental Chemistry, Faculty of Chemistry, University of Lodz, 163 Pomorska St., 90-236 Lodz, Poland; kamila.borowczyk@chemia.uni.lodz.pl; 4 Department of Inorganic and Analytical Chemistry, Faculty of Chemistry, University of Lodz, 12 Tamka St., 91-403 Lodz, Poland; dariusz.guziejewski@chemia.uni.lodz.pl

**Keywords:** tenofovir, voltammetry, glassy carbon, graphene oxide, silver amalgam

## Abstract

This paper discusses the electrochemical behavior of antiviral drug Tenofovir (TFV) and its possible applicability towards electroanalytical determination with diverse detection strategies using square-wave voltammetry. Namely, oxidation processes were investigated using glassy carbon electrode with graphene oxide surface modification (GO/GCE), while the reduction processes, related to the studied analyte, were analyzed at a renewable silver amalgam electrode (Hg(Ag)FE). Scanning electron microscopy imaging confirmed the successful deposition of GO at the electrode surface. Catalytic properties of graphene oxide were exposed while being compared with those of bare GCE. The resultant modification of GCE with GO enhanced the electroactive surface area by 50% in comparison to the bare one. At both electrodes, i.e., GO/GCE and Hg(Ag)FE, the TFV response was used to examine and optimize the influence of square-wave excitation parameters, i.e., square wave frequency, step potential and amplitude, and supporting electrolyte composition and its pH. Broad selectivity studies were performed with miscellaneous interfering agents influence, including ascorbic acid, selected saccharides and aminoacids, metal ions, non-opioid analgesic metamizole, non-steroidal anti-inflammatory drug omeprazole, and several drugs used along with TFV treatment. The linear concentration range for TFV determination at GO/GCE and Hg(Ag)FE was found to be 0.3–30.0 µmol L^–1^ and 0.5–7.0 µmol L^–1^, respectively. The lowest LOD was calculated for GO/GCE and was equal to 48.6 nmol L^–1^. The developed procedure was used to detect TFV in pharmaceutical formulations and patient urine samples and has referenced utilization in HPLC studies.

## 1. Introduction

**Figure 1:**
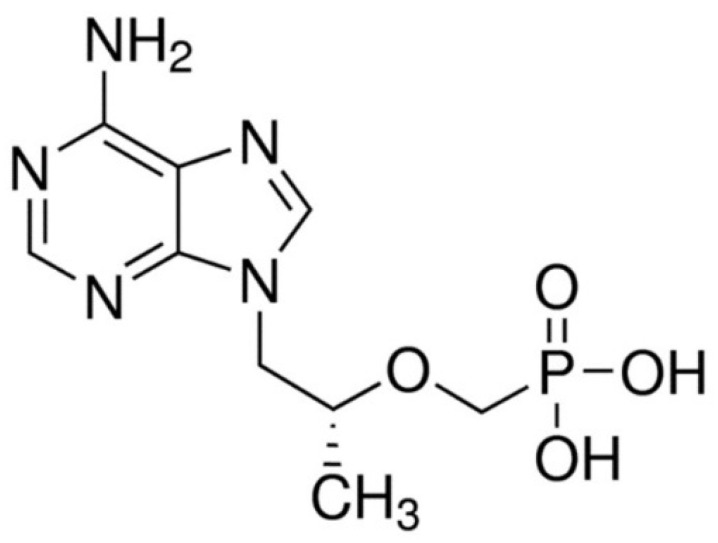
Structural formula of TFV.

According to the Center for Disease Control and Prevention (CDC) study from 2021, over a million people over the age of 13 were infected with the HIV virus in the USA, whereas, as reported by World Health Organization (WHO) and the United Nations program for Acquired Immunodeficiency Syndrome prevention (UNAIDS), in the entire world, the number of people with HIV/AIDS was estimated at the end of 2020 at approximately 38 million (3 million more than in 2013) [1]. AIDS is caused by infection with HIV-1 and HIV-2, which belong to the retrovirus family. HIV-1 is encountered worldwide in epidemic proportions, while the prevalence of HIV-2 is limited to West Africa [2]. Within 15 years, the mortality rate of patients infected with HIV decreased by at least 50%, which has led to the recognition of AIDS as a chronic disease instead of fatal one [3]. The number of deaths decreased sharply after HAART (Highly Active Antiretroviral Therapy) which was introduced in 1990. One of the groups of drugs to combat HIV infection includes NRTIs (nucleoside reverse transcriptase inhibitors), represented by Tenofovir (TFV; 1-(6-aminopurin-9-yl) propan-2-yloxymethylphosphonic acid; Figure 1) [4], which works by competing with deoxyadenozyno-5-triphosphate.

Many chromatographic and spectrophotometric methods for Tenofovir detection are known [5,6,7,8,9], while there are only few electrochemical ones [10,11,12,13,14]. Due to sensitivity, the cathodic potential window, and reproducibility, mercury electrodes are unique. Currently, there is a trend for the use of Green Chemistry, which relies on decreasing or even eliminating harmful compounds during analysis. Two groups of researchers have presented amalgam electrodes (based on dental amalgams and metal amalgamated powders) as environmentally friendly alternatives for mercury electrodes [15,16]. The next innovative type that has been introduced is the renewable silver amalgam film electrode (Hg(Ag)FE) developed by Kowalski and Baś [17,18]. Amalgam film electrodes designed by B. Baś were successfully used in various analytical applications, e.g., the determination of metal ions, vitamins, pesticides, and drugs [19,20,21,22,23,24]. Amalgam electrodes have many advantages, such as negative working potentials, long-term activity, good sensitivity, mechanical stability, low toxicity (compared with classical mercury electrodes), and quick pretreatment procedures. Both the mercury and amalgam electrodes operate at negative potentials and are not suitable for electrochemically active compounds oxidation. In the positive potential window, the best choice are solid electrodes such as glassy carbon electrodes (GCEs)—one of the most popular ones currently [25,26,27]—as they offer high chemical inertness, fast refreshment procedure, stability, and low costs. Various substances are used to modify GCEs’ surface to improve the catalytic properties, electroactive area, yield of the reaction, or the selectivity of the measurements [23,28,29,30]. Currently, one of the most popular modifiers are graphene and its derivatives. Graphene was discovered in 2004 [31] and, since then, it has been an object of many studies [32,33,34]. As a 2D one-atom sheet of carbon atoms in sp^2^ hybridization, graphene is used in many fields of science because of its various advantages, such as excellent electrical conductivity, high specific surface area, and thermal and electrical properties [32,35,36,37]. Graphene oxide (GO) is a structure comprising a single-layer graphite oxide with hydroxyl, epoxy, carbonyl, and quinine groups as functional groups. These groups generate very good electrochemical and mechanical properties as well as a strongly hydrophilic character, which results in good dispersibility in various solvents [38]. In the last decade, graphene oxide became very popular as a working electrode modifier [39,40,41,42]. The goal of this research was the comparative examination of the electrochemical behavior (oxidation and reduction on GO/GCE and Hg(Ag)FE, respectively) of Tenofovir. Moreover, in this study, a new method of TFV determination in drugs, spiked urine, and patient urine samples was developed. Received results were verified with high-performance liquid chromatography reference method.

## 2. Results and Discussion

### 2.1. Properties and Electrochemical Performance of the GO/GC Electrode

**Figure 2:**
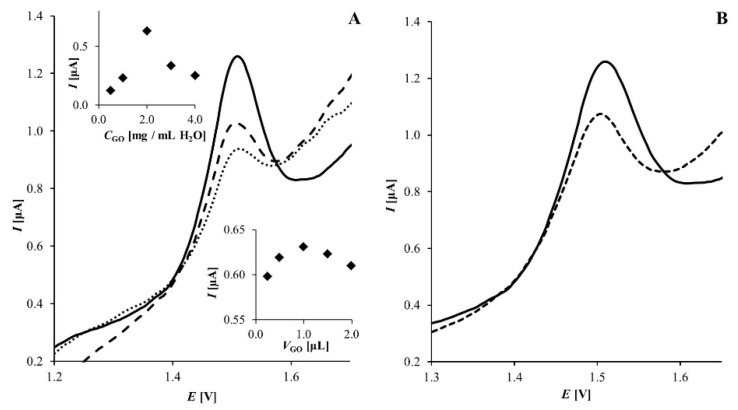
(**A**): TFV voltammograms recorded on GCE modified with GO suspensions. Concentrations of GO suspensions: 1 mg × mL^−1^ (dotted line), 2 mg × mL^−1^ (solid line), 3 mg × mL^−1^ (dashed line); inset left: influence of concentration of GO suspension on TFV peak current; inset right: influence GO suspension volume on TFV peak current; (**B**): Voltammograms of TFV recorded on bare GCE (dashed line) and GO/GCE (solid line).

Various modifying solutions based on nanomaterials were tested during introductory research. Among the tested nanosubstances were, inter alia, graphene oxides and carbon nanotubes. Conclusions about each modifier layer performance were drawn after comparisons were conducted between voltammograms obtained on modified and bare GCE. With respect to Tenofovir’s signal shape and height, the best results were obtained on GCE modified with a graphene oxide aqueous suspension (Figure 2A). Among different GO content in the suspension, the optimal response was recorded with 2 mg × mL^−1^ (Figure 2A, left inset). Next, various volumes of this suspension (0.5–4.0 µL) were dropped onto a GCE surface to investigate the influence of the modifier’s amount on TFV peaks. Based on the obtained results (Figure 2A, right inset), a volume equal to 1 µL was selected for subsequent studies. The comparison of optimized voltammogram with respect to the type and content of the modifier in relation to the bare glassy carbon electrode is presented in Figure 2B. The position of the Tenofovir oxidation signal is virtually unaffected, but its height increases when the electrode surface is modified. That fact is exhibited as an effect of much larger electroactive surfaces when compared to the bare GCE. What is worth noticing is also the changes in the overall shape of the voltammogram (here, cf. Figure 2B-lowered background current recorded at more positive potential) that cause improved separation from the current related to the oxidation of the electrode surface itself and, therefore, facilitate TFV signal characterization. The electrodes prepared by drop-casting 1 µL of 2 mg × mL^−1^ GO aqueous suspension (GO/GCE) were subjected to further studies.

**Figure 3:**
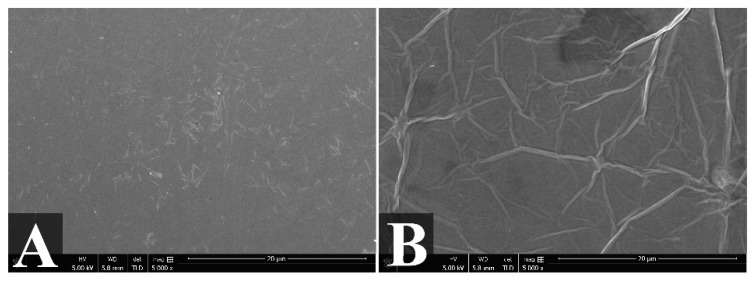
SEM images of bare glassy carbon electrode (**A**) and glassy carbon electrode modified by graphene oxide (**B**) at 5000× magnification.

Scanning electron microscopy was used to characterize GCE and GO/GCE surfaces. SEM analysis showed that the bare glassy carbon electrode exhibits smooth surfaces appropriate for the deposition of GO (Figure 3A). The GO flakes are uniformly attached to the substrate, creating a consistent coating without holes (Figure 3B). The obtained surface is rough due to the presence of noticeable wrinkles, which are typical for GO coatings. Wrinkles are formed due to water evaporation during the drying process of the GO dispersions [43]. Fabricated wrinkles increase the active surface area of a modified electrode. The morphology of the prepared GO coating is consistent with those previously reported [44].

**Figure 4:**
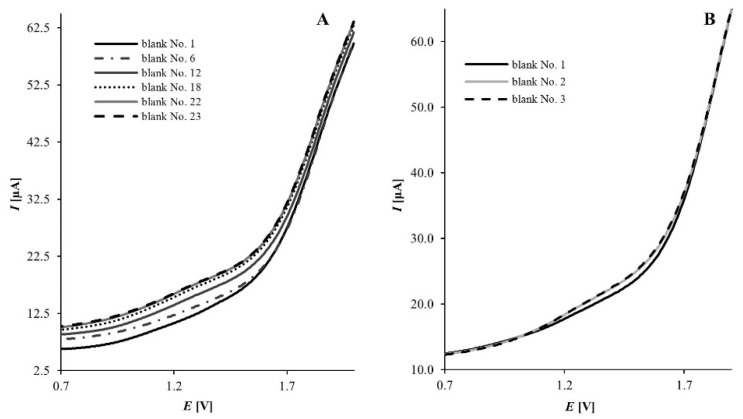
SW voltammograms recorded in supporting electrolyte on GO/GC electrode—(**A**): without cyclization; (**B**): after cyclization.

Then, the electroactive area of the GCE and GO/GCE was estimated from cyclic voltammograms of the hexacyanoferrate model redox system. For electrochemically reversible systems, peak current *I*_p_ is dependent on the electroactive area of the electrode (A): *I*_p_ = 2.69 × 10^5^ n^3/2^ A C * D^1/2^ ν^1/2^, where n is the number of electrons, C * is the concentration, *v* is the scan rate, and D represents the diffusion coefficient. From the slopes of *I*_p_ vs. ν ^1/2^, the electroactive surfaces of the GO/GC and GC electrodes were calculated and found to be 2.46 and 1.66 mm^2^, respectively. Therefore, the modified electrode had an increased surface area of 148%. Next, chronocoulometric measurements were employed to study the surface of the GO/GC and GC electrodes. On the basis of the saturated adsorption capacity (SAC) of bare and modified glassy carbon electrode, Q is proportionally dependent to t^1/2^ and may be described with following formula [45,46,47]: Q = [(2nFAcD^1/2^t^1/2^)/π^1/2^] +Q_ads_ + Q_dl_, where Q_ads_ is the charge of the oxidation of the adsorbed reagent, Q_dl_ is the double-layer charge, F is the Faraday constant, and the other symbols have meaning as described above. Using this equation, it was calculated that GO/GCE had an increased surface area of 156% in comparison to bare GCE. It has to be mentioned that the only one problem observed when working with GO/GCE was the constant parallel drift of the baseline in consecutive measurements (Figure 4A). Such instability in the baseline can cause problems or even make the determination process impossible. Hence, to improve baseline stability, GO/GCE was cycled in the supporting electrolyte in a polarization range of 1.5 V (from 0.5 V to 2.0 V). Voltammograms recorded for blanks after cyclization were improved (Figure 4B).

### 2.2. Electrochemical Behavior of Tenofovir

**Figure 5:**
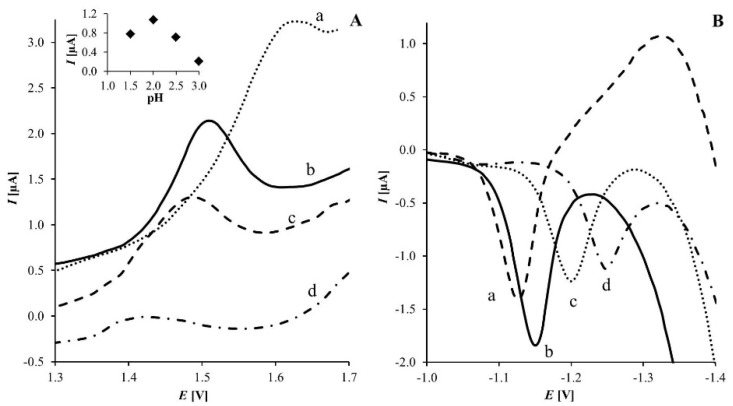
(**A**): SW voltammograms of TFV recorded on GO/GCE in a Britton–Robinson buffer at pH: a: 1.6, b: 2.0, c: 2.5, d: 3.0. Inset: influence of BR buffer pH on TFV peak current. (**B**): SW voltammograms of TFV recorded on Hg(Ag)FE in citrate phosphate buffer at pH: a: 2.0, b: 2.5, c: 3.0, d: 3.5.

The electrochemical behavior of Tenofovir was studied in a broad pH range as the acidity of the analyzed solution is one of the main factors influencing voltametric response of pharmaceuticals. On GO/GCE, preliminary studies were conducted in BR buffers in a pH range 1.6–8.0 (Figure 5A). As the highest signals were recorded in strongly acidic pH, the next examined supporting electrolytes were based on citrate–phosphate and chloride buffers, but good results were not observed. Due to this observation, the BR buffer with a pH of 2.0 was chosen for further studies. BR buffers were also used in preliminary studies conducted on Hg(Ag)FE. TFV signals were observed in a pH range of 2.0–6.0 with the highest ones recorded in pH 2.5. To check whether other buffers can offer signals of better morphology and height and taking into account that the highest TFV signals were observed in acidic BR buffers, two other buffers were examined: citrate buffer (pH 1.5–3.5) and citrate–phosphate buffer (pH 2.0–3.5). The best results were obtained in citrate phosphate buffers (Figure 5B). A citrate–phosphate buffer of pH 2.5 was selected for subsequent studies.

Of the plethora of electrochemical techniques that are available, cyclic voltammetry has become as a primary choice, especially for the characterization of undetermined reaction mechanisms. The performance ease and information richness available for such a simple current−potential response analysis provide an extensive analytical tool established for electrochemical response interpretation. Consequently, in the next step, cyclic voltammetry was used to characterize the nature of the TFV electrode reaction. Therefore, the influence of the scan rate (*ν*) on the TFV peak current (*I_p_*) at both electrodes was inspected.

**Scheme 1:**
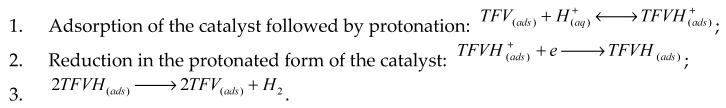
Suggested mechanism of Tenofovir electrochemical reduction.

**Figure 6:**
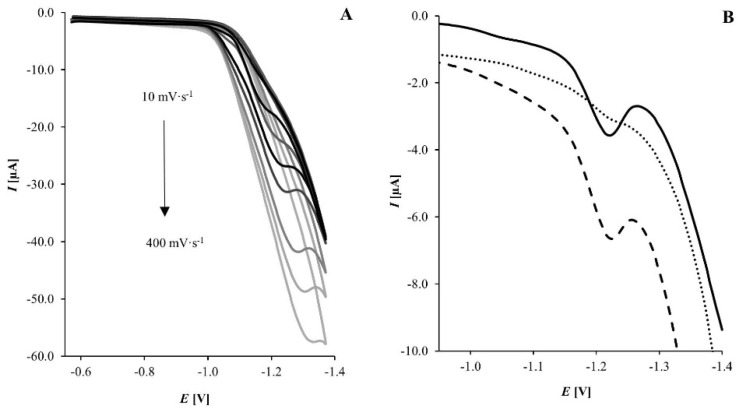
(**A**): Cyclic voltammograms of TFV (scan rates: 10, 30, 50, 100, 200, 300, and 400 mV·s^−1^) on Hg(Ag)FE; (**B**): SW voltammograms of TFV (*C*_TFV_ = 1.0 × 10^−5^ mol·L^−1^) (solid line) including components: forward (dashed line) and backward (dotted line) on Hg(Ag)FE.

A proposed mechanism for the TFV reduction on Hg(Ag)FE is provided in Scheme 1:

This mechanism suggests that the electrochemical reaction is an irreversible process. Irreversibility is confirmed by a few factors. First, as depicted in Figure 6A, no anodic peak was observed on the recorded cyclic voltammogram. The same evidence can be noted from the course of forward and backward components in square wave voltammetry (Figure 6B).

Second, linear dependence between the scan rate and Tenofovir peak currents together with the TFV peak potential shifted to more negative values upon the increase in scan rate, and this is typical for irreversible cathodic processes. Next, to examine if the electrode process is controlled by adsorption or diffusion, the dependence between log *I_p_* and log *ν* was constructed and this produced a slope of 1.05, which is consistent with the theoretical value of 1.0 and characteristic of electrode processes where adsorption takes place [48].

The facts presented above, together with drug chemical structure and the features of peak potential, point out to a CHE (catalytic hydrogen evolution reaction) mechanism, as indicated in Scheme 1, with a high probability. Theoretical and experimental studies of catalytic hydrogen evolution reaction taking place under the prevalence of electrocatalytically active compounds are reported in the literature [49]. Usually frequency effect during electrochemical studies within square-wave voltammetry of the compound possessing such electrocatalytic properties are suggested [24,50,51,52]. Having this in mind, the observation of the 5 × 10^–6^ M TFV peak current under the effect of square-wave frequency was performed. The received characteristic shapes, i.e., parabolic and exponential, of dependencies *I*_p_ = f(*f*) and *I*_p_*f*^−1^ = f(*f*), respectively, are consistent with the theory of CHE reaction mechanism. With the above-mentioned information, it can be rationalized that Tenofovir plays a role of in the CHE electrocatalyst and the overall mechanism is subjected to the equations provided in Scheme 1.

**Figure 7:**
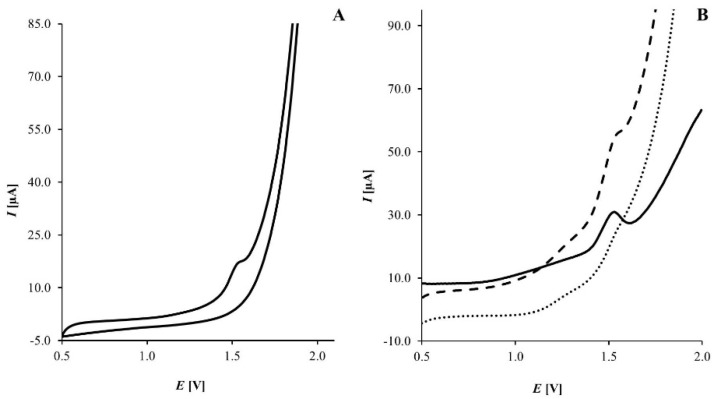
(**A**): Cyclic voltammogram of TFV recorded on GO/GCE; (**B**): SW voltammograms of TFV (solid line) including components: forward (dashed line) and backward (dotted line).

A similar irreversible behavior of Tenofovir was observed on GO/GCE (Figure 7A). As evident from the anodic part of the CV scan at GO/GCE, an oxidation process of TFV was observed at ca. 1.5 V. During the potential reverse, no changes in the residual current were noticed, suggesting completely irreversible process during Tenofovir electrochemical oxidation. Moreover, with increasing scan rate (from 10 mV s^− 1^ to 500 mV s^− 1^), TFV peak potentials shifted to more positive values, confirming the irreversibility of the electrode processes [48]. This was also proven with a square-wave voltammetry experiment where the components of the resulting current were of the same sign (Figure 7B). As observed, only the signal on the forward component can be observed, suggesting the occurrence of an oxidation process without the presence of any further electrochemical reduction received from the oxidized product, which would be observed during a backward potential pulse.

**Scheme 2:**
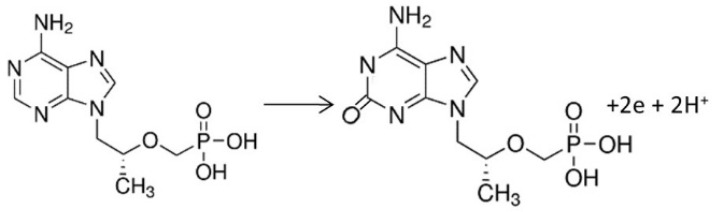
Suggested pathway of TFV oxidation.

Normally, relevant information about the mechanism of the electrode reaction (diffusion-controlled or adsorption-controlled) may be achieved from the relationship between peak current (*I_p_*) and scan rate (*v*) or its square root (*v^1/2^*). In the case of TFV, no reliable conclusions may be drawn from both dependencies. Such behavior was observed previously for compounds with similar chemical structure [53]. Next, the relationship between log *I_p_* and log *v* was examined. As it is known, values of slope for log *I_p_* vs. log *v* dependence are expected to be 1.0 for adsorption-controlled and 0.5 for diffusion-controlled reactions [48,54]. The mentioned dependence produced a slope of 0.72 (R^2^ = 0.9955), indicating that the oxidation current is of mixed nature. In the next step, the number of electrons involved in the electrooxidation of TFV was established. In CV experiments, the dependence of *E*_p_ on ln *ν* was described by equation *E*_p_ [V] = 0.0248 ln ν [V s^−1^] + 1.42. To calculate number of the electrons transferred in the rate determining step, Laviron’s equation, *E*_p_ [V] = *E^0^* + RTlnν/(1 − α)nF, (where α is the electron transfer coefficient and *E^0^* is the standard potential), was used. With α assumed as 0.5 [55], the number of electrons was calculated to be 2.07. In addition, the number of electrons was also calculated from the relationship between half-peak height potential and peak potential |*E*_p_ − *E*_p/2_| = 47.7/(αn) [56,57]. Considering α = 0.5, the number of electrons was n = 2.096 ≈ 2. Taking into account factors described above, we suggest that TFV oxidation occurs similarly to adenine oxidation [58,59,60,61,62]. Adenine oxidation involves two oxidation steps where the first one, 2e-process, is the rate-determining step and provides the 2-oxoadenine product [57,59,60]. As a Tenofovir molecule contains adenine moiety and the number of electrons transferred in the rate-determining step is consistent with those known for adenine, the possible pathway of Tenofovir oxidation is provided in Scheme 2.

### 2.3. Analytical Studies

#### 2.3.1. Calibration Curve

**Figure 8:**
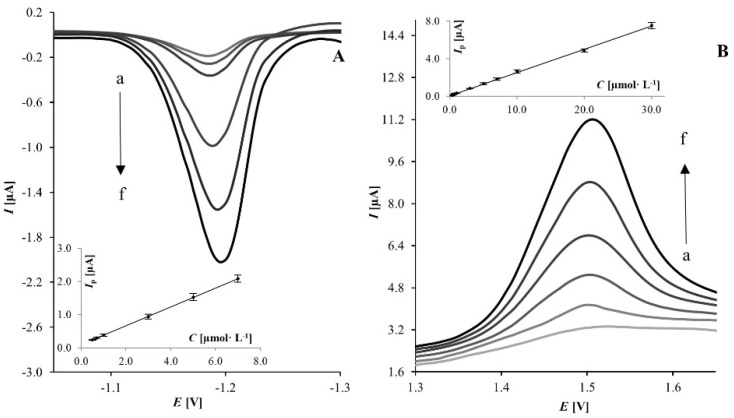
SW voltammograms of TFV. (**A**). On Hg(Ag)FE, a: 5.0 × 10^–7^, b: 7.0 × 10^–7^, c: 1.0 × 10^–6^, d: 3.0 × 10^–6^, e: 5.0 × 10^–6^, f: 7.0 × 10^–6^ mol L^–1^; (**B**). On GO/GCE, a: 5.0 × 10^–7^, b: 1.0 × 10^–6^, c: 5.0 × 10^–6^, d: 1.0 × 10^–5^, e: 2.0 × 10^–5^, f: 3.0 × 10^–5^ mol L^–1^; each inset: corresponding calibration curve.

The calibration curve for Tenofovir was evaluated under optimal conditions using both electrode reactions: reduction on Hg(Ag)FE and oxidation on GO/GCE. On silver amalgam electrode, the peak current increased linearly with TFV concentration from 5.0 × 10^–7^ to 7.0 × 10^–6^ mol L^–1^ (Figure 8A). When a graphene oxide modified GC electrode was used, the linear range started from 3.0 × 10^–7^ and ended at 3.0 × 10^–5^ mol L^–1^ (Figure 8B). For both methods, the limit of detection (LOD) was calculated, and this was equal to 1.35 × 10^−7^ mol L^–1^ and 4.86 × 10^−8^ mol L^–1^ for Hg(Ag)FE and GO/GCE, respectively. The calibration curve’s statistical parameters are listed in Table 1. As can be observed that the low values of coefficient of variation confirmed good reproducibility and precision of the proposed procedure.

For the sake of comparison, the linear range was also evaluated for bare GCE, and this was found to be 7.0 × 10^–7^–7.0 × 10^–6^ mol L^–1^. Taking into account the results described above, GO/GCE (rather than bare glassy carbon electrode) was chosen for subsequent experiments based on TFV oxidation. Considering other electrochemical methods of TFV determination in the literature and those presented in this manuscript (cf. Table 2), it can be concluded that the developed methodology has several advantages. The linear concentration range lays in the submicromolar region, while most of reported in other papers are micromolar and higher [10,11,12], and only one of the methods is more sensitive in this respect [13]. Moreover, it is worth mentioning that the method using oxidation at GO/GCE covers a full two-fold concentration range, which is not so common within other electrochemical methods of TFV detection. The analytical performance of the methods presented herein can be also emphasized by means of repeatability (<5%, cf. Table 1) and recovery (±5%). What is more is that the correctness of our methods was revealed not only in spiked but also in real samples as well, and it was confirmed with the reference chromatographic method. It is again worth stressing the possibility of diverse detection strategies (based on oxidation or reduction reaction) that will be able to deal with the possible unpredicted effects of non-studied interferents or the matrix problem more appropriately.

#### 2.3.2. Analysis of Pharmaceutical Formulations

The developed procedure was successfully used for the determination of Tenofovir in pharmaceutical formulations: Viread and Tenofovir disoproxil Teva. Samples prepared from tablets were made as described in Section 3.4. To avoid matrix effects, the standard addition method (SAM) was used. Unfortunately, the usage of SAM was insufficient to eliminate the matrix effect with GO/GC electrodes, where substances present in tablets made its determination impossible (recovery obtained for GO/GCE was around 40% for both formulations). To eliminate the possible negative influence of graphene oxide, experiments were repeated on bare GC electrodes—good results were not obtained. These difficulties were not observed in the experiments with silver amalgam electrodes. Recovery was equal to 97.5% and 104.4% for Viread and Tenofovir disoproxil Teva, respectively, confirming that the determination of Tenofovir was sensitive and effective. The results are presented in Table 3.

#### 2.3.3. Analysis of Urine Samples

The developed procedure was used to detect Tenofovir in spiked urine samples. Urine samples (both spiked and patient’s) analysis was impossible on Hg(Ag)FE due to the negative influence of substances present in the urine matrix. Results of spiked urine samples analysis obtained on GO/GCE are listed in Table 4. Good accuracy of the method was confirmed by the average percent recoveries.

Moreover, the developed method was verified with real samples analysis (Table 5). Urine samples were taken from patients after 12h from TFV intake (one tablet of Tenofovir disoproxil Teva). During preliminary studies with patient’s urine samples, the electrochemical behavior of urine matrix was examined. It was stated that the urine matrix produces two voltametric peaks (*E*_1_ = 0.65V and *E*_2_ = 0.95V). As the observed signals are out of potential range used for TFV determination, it was concluded that the urine matrix did not interfere with the voltametric analysis of TFV on GO/GCE. The recovery experiment for TFV in the collected urine samples has been performed by the addition of 200 µL of urine sample to 9.8 mL of the supporting electrolyte. TFV concentrations have been measured via a standard addition protocol. The same samples were analyzed by the reference HPLC method, as described in the Experimental Section. In order to statistically assess the compliance of the results obtained with SWV and HPLC methods, the student’s *t*-test was applied. The calculated values of *t* (1.697 and 2.035) were compared with the tabular value of *t_cr_* (2.776). The calculated values were lower than the tabular value; therefore, the obtained results do not differ statistically significantly. Thus, it can be stated that the results obtained with both methods were consistent and consequently confirmed the utility of voltametric method.

#### 2.3.4. Selectivity of the Method

The influence of substances that could be present in biological samples was investigated. The effect of possible interfering agents (IA), such as ions and organic molecules, was examined, as described in Section 3.5. In the examined concentration range, interference was not observed (the percentage of the peak current change were proved to be less than 10% in comparison to those obtained with uncontaminated TFV solutions) for ascorbic acid, ibuprofen, acetylsalicylic acid, glucose, fructose, sucrose, glycine, lysine, and phenylalanine; and the cations Ca^2+^, Mg^2+^, Cd^2+^, Fe^2+^, Fe^3+^, Na^+^, and K^+^ on both Hg(Ag)FE and GO/GCE. Lactose interfered electrochemical determination of TFV on GO/GCE, while omeprazole and metamizole when Hg(Ag)FE was used. Moreover, as GO/GCE was used for urine samples analysis, three drugs (lopinavir, emtricitabine, or ritonavir), which may be used in patient treatment together with TFV, were examined as possible interferents. A negative influence of these compounds on the electrochemical determination of TFV on GO/GCE was not observed.

## 3. Materials and Methods

### 3.1. Apparatus

Electrochemical measurements were performed using a µAutolab type III potentiostat (EcoChemie, Utrecht, Netherlands) connected to PC with GPES v. 4.9 software. An M164 stand (MTM Anko Instruments, Krakow, Poland) with a standard three electrode system comprised of Hg(Ag)FE (AGH University of Science and Technology, Cracow, Poland), bare GCE or graphene oxide modified GCE (GCE from BASI, IN, USA, geometric area 7.1 mm^2^) as the working electrode, Ag/AgCl as the reference electrode, and platinum wire as the counter electrode. Hg(Ag)FE was employed for the electrochemical reduction of Tenofovir, while GCE and GO/GCE were used as working electrodes in TFV electrooxidation. The Hg(Ag)FE electrode has an adjustable geometric area, which can be easily changed from 1.5 to 12.0 mm^2^; in this phase, a study area of 8.0 mm^2^ was used. The surface of Hg(Ag)FE was renewed before each experiment. GCE’s surface was cleaned by polishing pretreatment with 0.05 μm alumina and then carefully washed with double distilled water, immersed in water, sonicated for 3 min, and dried in air.

Subsequently, a water suspension of graphene oxide was kept in an ultrasonic bath for 5 min. GO/GCE was prepared by dropping 1.0 µL of the GO suspension directly onto the electrode’s surface and leaving it to dry. A scanning electron microscope (NovaNano SEM 450, FEI, Hillsboro, OR, USA) with a through lens detector (TLD) in immersion mode was used in the imaging of the working electrode surfaces. Images were acquired at a magnification of 5000×. HPLC analyses were performed on a 1220 Infinity LC system from Agilent (Santa Clara, CA, USA) equipped with a binary pump integrated with a two-channel degasser, autosampler, column oven, and DAD detector. All chromatographic separations were completed on the ZORBAX SB C-18 (150 × 4.6 mm, 5 µm) column delivered by Agilent Technologies, (Waldbronn, Germany). The control of the 1220 Infinity HPLC system was achieved by application of the OpenLAB CDS ChemStation Edition.

### 3.2. Measurement Procedure

Voltammetric measurements were made as follows: The supporting electrolyte was placed in the cell and the solution was deaerated with argon for 600 s (deoxygenation was not conducted with GCE and GO/GCE). When voltammogram of the blank was recorded, the appropriate volumes of Tenofovir were added to electrochemical cell. Then, the solution was deoxygenated for 10 s, and sample voltammogram was recorded. For Hg(Ag)FE and GO/GCE, the optimal SW parameters were as follows: amplitude 30 mV, step potential 4 mV, frequency 25 Hz and amplitude 30 mV, step potential 7 mV, and frequency 30 Hz, respectively. In the HPLC reference method for the separation of the analyte, a gradient elution was used (0–6 min 2–4% A; 6–7 min 4–2% A; 7–8 min 2% A), where the following is the case: (A) was acetonitrile, and (B) was 0.015 mol L^−1^ phosphate buffer with a pH of 7.4. The column temperature was 25 °C, the flowrate was 1 mL min^−1^, and the detector wavelength was 260 nm. The retention time of the analyte was 4.1 min. The total time of the analysis was 10 min, with 8 min of analysis and 2 min of column reconditioning. The identification of the peak of the analyte was based on the comparison of retention time with a corresponding set of data obtained for the authentic compound.

### 3.3. Materials and Solutions

All chemicals used were of reagent grade (Sigma Aldrich, Saint Louis, MO, USA) and were not further purified. Standard stock solutions of Tenofovir (1.0 × 10^–3^ mol × L^−1^) were prepared by direct dissolution in water. Working solutions were prepared by a serial dilution of the stock solution. Commercially available formulations—Viread (Gilead, Foster City, CA, USA) and Tenofovir disoproxil Teva (Teva, Warszawa, Poland)—were purchased from a local pharmacy in Lodz (Poland). The supporting electrolytes were as follows: citrate–phosphate, citrate, Britton-Robinson (BR) and chloride buffers. All solutions were prepared with double distilled and deionized water.

### 3.4. Analysis of Pharmaceutical Formulations and Urine Samples

For tablet solutions, six tablets of Viread/Tenofovir disoproxil Teva were weighed and the average mass per tablet was determined. An appropriate weighed portion of a tablet powder was dissolved to produce a 1.0 × 10^–3^ mol L^–1^ solution. Tablet solutions were filtered through a Millipore filter to separate insoluble excipients. Urine was taken from patients treated with TFV and from healthy volunteers. During first morning, urine samples were obtained from patients and volunteers and analyzed without delay or kept at −80 °C. To prepare spiked urine samples, accurately measured aliquots of TFV solutions were placed into volumetric flasks and filled up to volume with urine obtained from healthy volunteers. Urine solutions (both real and spiked samples) were analyzed without sample pretreatment or extraction steps. For the chromatographic determination of Tenofovir, 450 µL of phosphate buffer (0.015 mol L^−1^; pH 7.4) was added to 50 µL of urine. Ten microliter of final analytical solutions was injected into the chromatographic system. The nominal contents of TFV in all described samples were calculated using a standard addition method.

The described studies were conducted in accordance with the Declaration of Helsinki and accepted by the Ethical Committee of the University of Łódź. All participants provided informed consent for participation in these studies.

### 3.5. Interference Studies

The influence of possible interfering agents (IA), including ions and organic molecules, was examined. First, 50 μL or 100 μL (for Hg(Ag)FE and GO/GCE, respectively) of stock solution of TFV was added to the cell with the supporting electrolyte and the SW voltammogram of TFV was recorded (the final TFV concentration in the cell was 5.0 × 10^−6^ mol L^−1^ and 1.0 × 10^−5^ mol L^−1^ for Hg(Ag)FE and GO/GCE, respectively). Next, each IA was added at concentrations of 2.5 × 10^−7^, 5.0 × 10^−7^, 1.0 × 10^−6^, 2.5 × 10^−6^, 5.0 × 10^−6^, 1.0 × 10^−5^, 2.5 × 10^−5^, 5.0 × 10^−5^, and 1.0 × 10^−4^ mol L^−1^ for Hg(Ag)FE; and 5.0 × 10^−7^, 1.0 × 10^−6^, 2.0 × 10^−6^, 5.0 × 10^−6^, 1.0 × 10^−5^, 2.0 × 10^−5^, 5.0 × 10^−5^, 1.0 × 10^−4^, and 2.0 × 10^−4^ mol L^−1^ for GO/GCE to the cell with *Tenofovir*, and SW voltammograms were registered after each addition of IAs. The final concentration ratios *(cIA:cTFV)* were as follows: 1:20, 1:10, 1:5, 1:2, 1:1, 2:1, 5:1, 10:1, and 20:1. Moreover, lopinavir, emtricitabine, and ritonavir were tested on GO/GCE, as these drugs are commonly used together with Tenofovir. In this case, the concentration ratios *(cIA:cTFV)* were chosen on the basis of dosages commonly used in such combination therapy.

### 3.6. Validation of the Method

A calibration curve (y = ax + b) was constructed as a dependence between TFV peak current (*I*_p_, A) and concentration (C, mol × L^–1^). The limit of detection (LOD) and limit of quantification (LOQ) were calculated from 3*sa*^–1^ and 10*sa*^–1^, where *a* is the slope of the calibration curve, and *s* is the standard deviation of the peak heights (five runs; for lowest TFV concentration from calibration curve range) [41]. Repeatability was calculated after five measurements at the same Tenofovir concentration. The reproducibility was estimated on the basis of five measurements on different days [63]. Repeatability was measured using the same GO/GC electrode, while each measurement used to calculate reproducibility was registered using GO/GCE with new modifier layer. In the case of Hg(Ag)FE, the electrode surface was renewed before each measurement, due to a specific construction of the electrode. To evaluate recovery (R), the R = (found/added) × 100% formula was used. Confidence interval and coefficient of variation were calculated from the equations following: t(S/n^1/2^), *p* = 95%, n = 5 and CV = (*SD/ave*) × 100% (*ave*—average from measured values; *SD*—standard deviation between those values), respectively. Seven-point calibration plots were used to test the linearity of the HPLC method. Each concentration was assessed in four replicates. A linear relationship was expressed as a peak area and TFV concentration correlation in the range from 0.01 to 0.30 mg mL^−1^. The linear equation was expressed as y = 2020x + 0.4745, while the correlation coefficient was R^2^ = 0.9996. According to the validation procedures, to test the precision and accuracy of the method, samples spiked with known amounts of TFV were analyzed in triplicate. Precision was defined as the relative standard deviation (RSD). Accuracy was established as the percentage of analyte recovery calculated as a difference between the resultant amount and the true/added amount of TFV. The mean RSD (%) and mean recovery (%) were 3.41 and 99.29, respectively. To compare results obtained via the SWV and HPLC methods, Student’s *t*-test was used. In the first step, the *t* value was calculated using *t* = [|*ave*_SWV_−*ave*_HPLC_|/(*SD*^2^_SWV_ + *SD*^2^_HPLC_)^1/2^] n^1/2^, where *ave, SD*, and n have the same meaning as stated above. In the next step, the tabular value of *t*_cr_ was checked for the chosen statistical significance (0.05) and appropriate degrees of freedom. The calculated *t* (for each series) was compared with *t*_cr._

## 4. Conclusions

A simple and efficient SWV method for TFV determination based on Hg(Ag)FE and GO/GCE usage was established. Under the same experimental conditions, an electrode modified by graphene oxide showed better electrochemical activity toward TFV than bare GCE. The SEM technique was used to show differences in the morphology of GCE and GO/GCE surfaces. Electroanalytical measurements revealed that modified electrode exhibit much larger electroactive surface when compared to bare GCE. For analytical studies, Hg(Ag)FE and GO/GCE were chosen. TFV produced a well-defined reduction peak at about –1.2 V on the silver amalgam film electrode and, on graphene oxide-modified glassy-carbon electrode, an oxidation peak at about 1.5 V. Both the silver amalgam film electrode and graphene oxide-modified glassy carbon electrode were successfully used for the quantitative detection of Tenofovir. Using SWV, the current response of TFV increased linearly with increasing concentrations from 0.5 to 7.0 µmol L^–1^ and from 0.3 to 30.0 µmol L^–1^ on Hg(Ag)FE and GO/GCE, respectively. LODs were found to be 1.35 × 10^−7^ and 4.86 × 10^−8^ mol L^–1^ for Hg(Ag)FE and GO/GCE, respectively. It was stated that Hg(Ag)FE can be applied for direct TFV determination in pharmaceutical formulations, where GO/GCE enables the determination of TFV in spiked and patients urine samples, indicating that the developed voltametric procedure can be applied to routine analysis. According to this, the carbon electrode modified by graphene oxide may have wider application in studies of Tenofovir; nonetheless, the merits of the both methods are as follows: low cost, easy construction and storage, potential for miniaturization, and fast analysis. In comparison to previous reports on the electrochemical determination of Tenofovir, the herein proposed procedure offers significantly extended linear responses toward the analyte. Furthermore, we have demonstrated that the GO/GC electrode may also be used in real samples analysis, i.e., patients’ urine.

## Figures and Tables

**Table 1:** Regression parameters for quantitative determination of TFV on Hg(Ag)FE and GO/GCE.

	Hg(Ag)FE	GO/GC
Linear range (mol L^–1^)	5.0 × 10^–7^–7.0 × 10^–6^	3.0 × 10^–7^–3.0 × 10^–5^
LOD (mol L^–1^)	1.35 × 10^−7^	4.86 × 10^−8^
LOQ (mol L^–1^)	4.48 × 10^−7^	1.62 × 10^−7^
R^2^	0.9999	0.9992
Reproducibility of the peak current, CV (%)	6.6	6.7
Reproducibility of the peak potential, CV (%)	2.5	4.3
Repeatability of the procedure, CV (%)	3.9	3.8

**Table 2:** Methods for electrochemical determination of TFV.

Method	Surface Modification If Applicable and Electrode Type	Sample Type	Linear Concentration Range [mol L^–1^]	LOD [mol L^–1^]	LOQ [mol L^–1^]	Reference
adsorptive stripping differential pulse and square-wave voltammetry	bare GCE	pharmaceutical formulation	6.0 × 10^−7^–6.0 × 10^−5^ both	1.02 × 10^−7^ and 8.40 × 10^−8^, respectively	3.39 × 10^−7^ and 2.80 × 10^−7^, respectively	[14]
adsorptive stripping square wave voltammetry	benzalkonium chloride coated AgNP on GCE	pharmaceutical dosage form	6.0 × 10^−8^–1.0 × 10^−6^	2.39 × 10^−9^	7.96×10^−9^	[13]
cathodic adsorptive stripping square-wave voltammetry	hanging mercury drop electrode	human plasma and tablet formulation	1.7 × 10^−6^–1.7 × 10^−5^	-	1.3×10^−6^	[11]
differential pulse voltammetry	nickel-cobalt sulphide decorated graphene quantum dots on GCE	pharmaceutical formulation, spiked urine	5.0 × 10^−6^–1.8 × 10^−5^	1.21 × 10^−8^	-	[10]
square wave voltammetry	boron-doped diamond electrode	pharmaceutical formulation	5.0 × 10^−6^–1.0 × 10^−4^	1.9 × 10^−6^	5.6 × 10^−7^	[12]
square wave voltammetry	renewable silver amalgam electrode	pharmaceutical formulations	5.0 × 10^–7^–7.0 × 10^–6^	1.35 × 10^−7^	4.48 × 10^−7^	This work
square wave voltammetry	graphene oxide on GCE	spiked and real patients urine	3.0 × 10^–7^–3.0 × 10^–5^	4.86 × 10^−8^	1.62 × 10^−7^	This work

**Table 3:** Results of pharmaceutical formulation’s analysis.

Pharmaceutical Formulation	Declared (mg)	Found (mg)	Recovery (%)
Viread	245.0	238.8 ± 14.1	97.5
Tenofovir disoproxil Teva	245.0	255.8 ± 14.3	104.4

**Table 4:** Results of spiked urine samples analysis.

Declared (μmol·L^−1^)	Found (μmol·L^−1^)	CV (%)	Recovery (%)
10.0	10.3 ± 0.9	9.5	103.0
50.0	47.7 ± 4.0	9.7	95.5
100.0	97.5 ± 7.6	8.9	97.5

**Table 5:** Results of patient’s urine analysis.

Patient	TFV Content µg × mL^−1^	
	SWV	HPLC
I	98.4 ± 8.1	86.8 ± 7.8
II	88.7 ± 7.5	80.1 ± 6.5

## Data Availability

The data presented in this study are available upon request from the corresponding author.

## Sample Availability

Not available.
